# Double Tibic Peroneal Neurectomy

**Published:** 1901-05

**Authors:** 


### DOUBLE TIBIC PERONEAL NEURECTOMY.

A correspondent, in referring to the coming publication by W. R. Jenkins on "Double Tibic Peroneal Neurectomy" for the relief of spavin and lameness, by Dr. W. E. A. Wyman, says that the latter takes issue with Bossi, who gave this operation to the profession as to anatomical descriptions, and refers to alleged imperfect and improper descriptions of the deep peroneal nerve by Chauveau, Strangeway; also the erroneous description by Williams in his translation of Pfeiffer. That current literature has occasionally conveyed some faulty impressions of a few cases operated upon and made questionable deductions therefrom. The book will record a large number of operations covering a period of three years, and the results obtained, with due consideration of the advantages and disadvantages of the operation.

